# Screening of the Medicines for Malaria Venture Pandemic Response Box for Discovery of Antivirulent Drug against Pseudomonas aeruginosa

**DOI:** 10.1128/spectrum.02232-22

**Published:** 2022-10-27

**Authors:** Markéta Macho, Subhasish Saha, Grzegorz Konert, Avik Banerjee, Daniela Ewe, Pavel Hrouzek, Petra Urajová, Kumar Saurav

**Affiliations:** a Laboratory of Algal Biotechnology, Institute of Microbiology of the Czech Academy of Sciences-Center Algatech, Třeboň, Czech Republic; b University of South Bohemia, Faculty of Science, České Budějovice, Czech Republic; c Laboratory of Photosynthesis, Institute of Microbiology of the Czech Academy of Sciences—Center Algatech, Třeboň, Czech Republic; Indian Institute of Science Bangalore

**Keywords:** *Pseudomonas aeruginosa*, quorum sensing inhibition, antivirulence activity, biofilm inhibition, Pandemic Response Box

## Abstract

Resistance development and exhaustion of the arsenal of existing antibacterial agents urgently require an alternative approach toward drug discovery. Herein, we report the screening of Medicines for Malaria Venture (MMV) Pandemic Response Box (PRB) through a cascade developed to streamline the potential compounds with antivirulent properties to combat an opportunistic pathogen, Pseudomonas aeruginosa. To find an agent suppressing the production of P. aeruginosa virulence factors, we assessed the potential of the compounds in PRB with quorum sensing inhibitory activity. Our approach led us to identify four compounds with significant inhibition of extracellular virulence factor production and biofilm formation. This provides an opportunity to expand and redirect the application of these data sets toward the development of a drug with unexplored target-based activity.

**IMPORTANCE** The rise of drug-resistant pathogens as well as overuse and misuse of antibiotics threatens modern medicine as the number of effective antimicrobial drugs steadily decreases. Given the nature of antimicrobial resistance development under intense selective pressure such as the one posed by pathogen-eliminating antibiotics, new treatment options which could slow down the emergence of resistance are urgently needed. Antivirulence therapy aims at suppressing a pathogen’s ability to cause disease rather than eliminating it, generating significantly lower selective pressure. Quorum sensing inhibitors are thought to be able to downregulate the production of virulence factors, allowing for smaller amounts of antimicrobials to be used and thus preventing the emergence of resistance. The PRB constitutes an unprecedented opportunity to repurpose new as well as known compounds with cytotoxicity and *in vitro* absorption, distribution, metabolism and excretion (ADME) profile available, thus shortening the time between compound discovery and medicinal use.

## OBSERVATION

The emergence of antimicrobial resistance (AMR) due to current strategies to battle against infectious diseases is recognized as a major threat and considered a slow-moving pandemic that is worsening every day. The rise in AMR infection is inevitable due to the use and misuse of antibiotics and prolonged hospital stay during the ongoing COVID-19 pandemic, making patients more vulnerable to attack by opportunistic pathogens (1). This fact provokes the urgent need to search for a new drug for instant use in case of an outbreak. A drug repurposing approach, consisting of screening known drugs to discover an already studied drug, sometimes already approved for clinical use, with a new mode action, can be used to fulfill this demand. This will strengthen our preparedness and fulfill our demand of fully exhausting the arsenal of antimicrobial drugs in use (2). The majority of pathogens causing nosocomial infections regulate their virulence factor production via quorum sensing (QS) (3). One of the well-studied opportunistic pathogens is Pseudomonas aeruginosa, which causes acute and chronic pulmonary infection among cystic fibrosis patients, with intrinsic resistance to multiple classes of antibiotics, including macrolides (4). P. aeruginosa uses QS systems to regulate production of virulence factors organized in a hierarchical manner (5). The top of canonical QS system includes the Las system consisting of LuxI-type synthases (LasI) which produces *N*-(3-oxododecanoyl)-l-homoserine lactone (OC_12_-HSL). Once the bacterial density reaches its quorum, the OC_12_-HSL/LasR complex positively activates the transcriptional regulation of *rhlR*, *rhlI*, *lasI*, and other virulence genes that are part of its regulon (6), followed by positive regulation of PqsR, thus producing the Pseudomonas quinolone signals (7, 8). Additionally, QS can contribute to behaviors that enable bacteria to resist antimicrobial compounds, e.g., biofilm development (9, 10). Selectively interfering with QS systems is one of the novel strategies targeted at disarming virulent opportunistic pathogens such as P. aeruginosa (11, 12). Among several known QS inhibitors (QSIs), furanone C30, penicillic acid, or various homoserine lactone derivatives, azithromycin is one of the first antivirulence-based drugs evaluated for their potential for treating bacterial infections in patients in a randomized clinical trial (13, 14). In several instances, QSI agents were also searched from a library of approved drugs, which led to the discovery of clinically approved drugs with new activity, such as niclosamide, clofoctol, and albendazole (15).

Here, we report the screening of the Medicines for Malaria Venture (MMV) Pandemic Response Box (PRB) for QSI activity and to determine the possibility to discover compounds with antivirulent activity among clinically approved drugs. In the current study, we used two widely used bioluminescence-based QS reporters constructed using the *luxCDABE* operon, derived from Photorhabdus luminescens, controlled by the PluxI gene together with the Vibrio fischeri *luxR*/*lasR* DNA fragment for evaluating QSI activity (16). When transformed in Escherichia coli, it emits luminescence in response to the exogenous addition of acyl homoserine lactones (AHLs; short [C_6_ to C_8_] acyl side chain length for E. coli pSB401 and long [≥C_10_] acyl side chain for E. coli pSB1075) (17).

**FIG 1:**
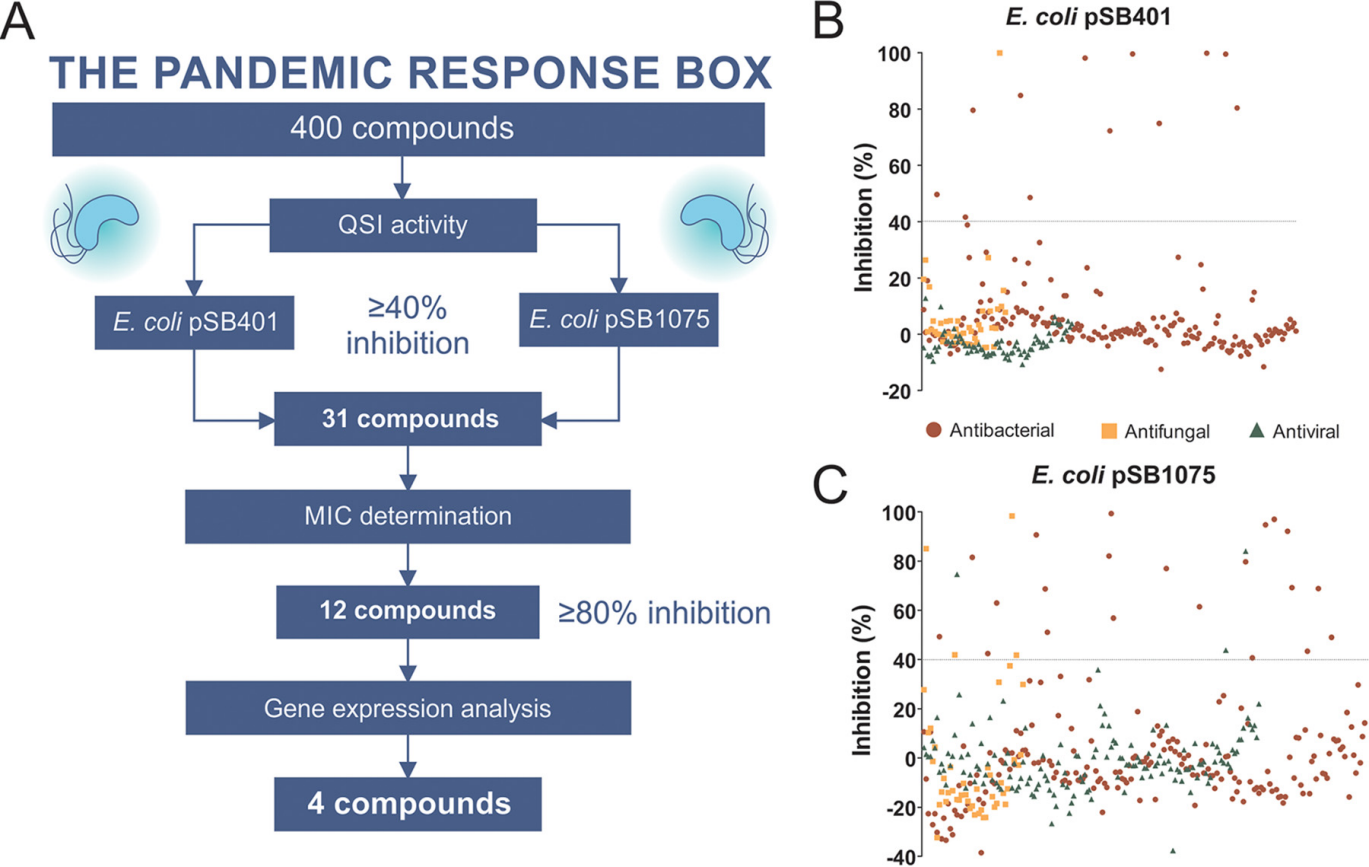
(A) Screening cascade of the MMV Pandemic Response Box for selecting the compounds for antivirulence activity. The criteria for each decision point are mentioned, followed by the number of active compounds that passed the criteria. (B and C) Screening of 400 compounds in the PRB against two bioreporter strains, E. coli pSB401 (B) and E. coli pSB1075 (C), to evaluate their quorum sensing inhibitory activity at 20 μM. Hits were selected based on ≥40% inhibition observed at 20 μM concentration as indicated by dotted line.

**FIG 2:**
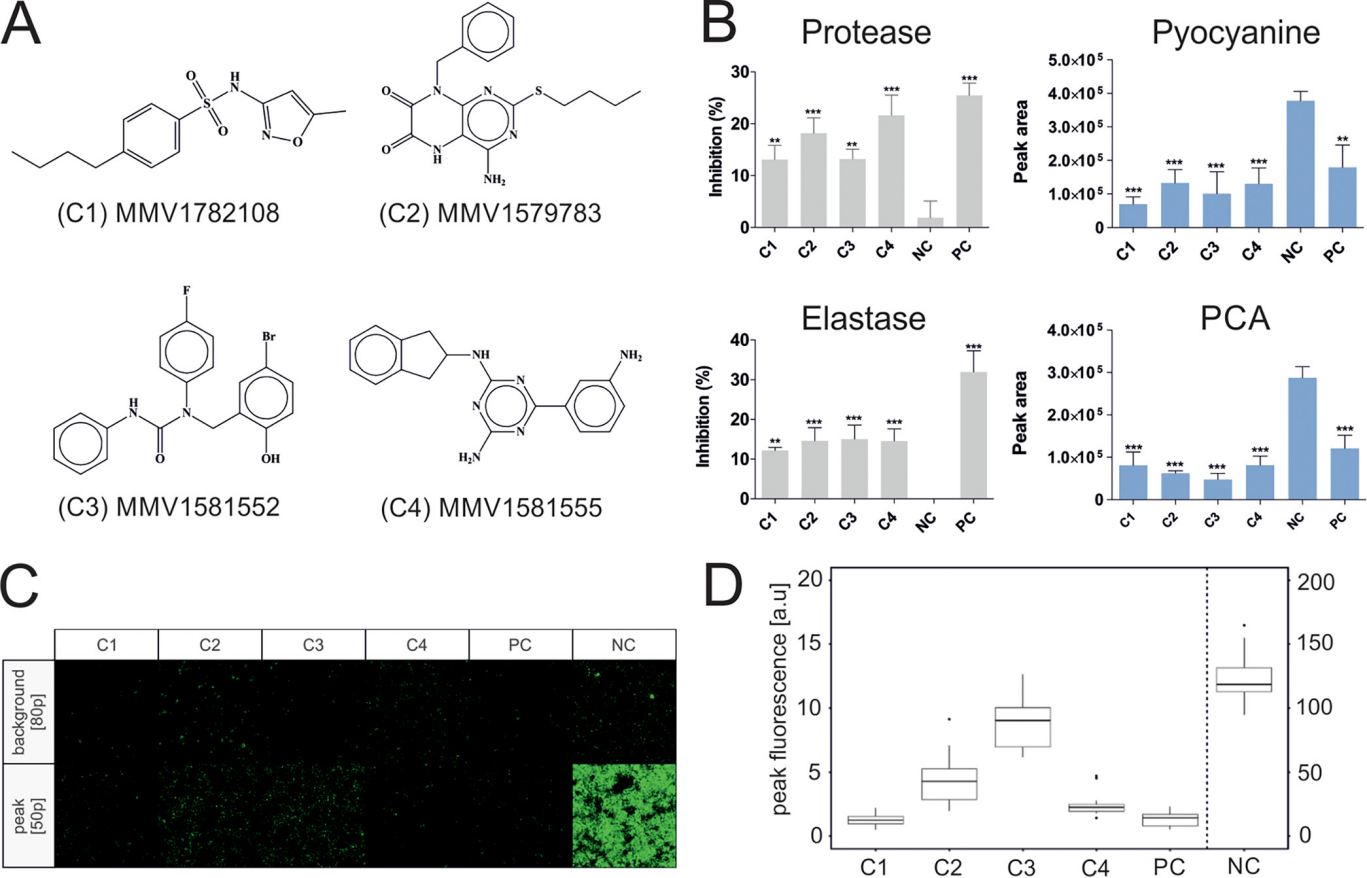
(A) Structures of all the four most potent compounds selected for antivirulence activity. (B) Protease, elastase, pyocyanin, and phenazine-1-carboxylic acid (PCA) inhibitory activity of compounds (C1 to C4) and furanone C-30 as a positive control (PC) in previously determined sub-MICs (Table 1) after 40 h of growth at 37°C. Error bars represent SD (*n* = 3). Data were analyzed using one-way analysis of variance (ANOVA) followed by Dunnett’s multiple-comparison test (*, *P* < 0.05; **, *P* < 0.01; ***, *P* < 0.001). (C and D) Quantum dot (QD) fluorescence-based biofilm inhibitory assay. The highest intensity peak corresponds with the count of QDs trapped in the biofilm layer. Tested sample values are close to positive-control values, while negative-control values are 10 times higher. All samples (C1, C2, C3, C4, and PC) are statistically significantly different from the negative control (*P* < 0.05). The statistical analysis was carried out with R 3.6.2 in RStudio 1.2.5033. Data were analyzed with one-way ANOVA followed by *post hoc* Tukey test.

The PRB is a collection of 400 structurally diverse compounds stratified by antibacterial, antiviral, and antifungal activities (201, 153, and 46 compounds, respectively) (18). A screening cascade was developed to streamline the potential compounds with significant QSI activity by filtering them stepwise (Fig. 1A). Two parallel screenings were designed against two bioluminescence-based bioreporter strains, each activated by their respective cognate autoinducers (irrespective of any antimicrobial activity) (17). Hits were identified with a relatively lenient but inclusive cutoff of ≥40% inhibition at a 20 μM concentration. A total of 31 compounds showed inhibitory activity against at least one of the bioreporter strains used (Fig. 1B and C): 13 against E. coli pSB401 and 28 against E. coli pSB1075; that is, 5.97% of the antibacterial and 2.17% of antifungal compounds tested showed activity against pSB401. Similarly, 11.44% of the antibacterial, 6.52% of antifungal, and 1.3% of antiviral compounds tested showed activity against E. coli pSB1075. None of the antiviral compounds showed any inhibitory activity against pSB401. Further, compounds showing growth inhibitory activity against both the bioreporter strains and P. aeruginosa PAO1 at a 20 μM concentration (Table 1) were filtered out to avoid any artifact discrepancy between bioluminescence and growth inhibition. Noninhibitory compounds with a potency filtration together with gene expression analysis (*lasR* and *rhlR*) in conjunction with the sub-MIC led us to select four most potent compounds, C1 to C4 (Fig. 2A; see also Fig. S1 and supplemental methods in the supplemental material). Our stringent profiling assay together with gene expression analysis in the cascade ensured selection of the most potent compound for the antivirulence study. Further, a time-kill kinetics study was also performed for all the four selected compounds (C1 to C4) to rule out any possibility of inhibitory activity, and it was observed that none of the selected compounds showed any inhibitory activity against P. aeruginosa PAO1 even at 40 μM after 48 h of growth (Fig. S3). Recently, a similar screening cascade was used for the discovery of an antimalarial drug and suggested a specific protein target for drug development (19).

**TABLE 1:** Compounds exhibiting inhibition against E. coli pSB401 and E. coli pSB1075 at a 20 μM concentration and their MIC and sub-MIC values against P. aeruginosa PAO1^*a*^

Activity	Compound	Inhibition (%) at 20 μM		P. aeruginosa	
		pSB1075	pSB401	MIC (μM)	Sub-MIC^*b*^ (μM)
Antibacterial	**MMV1579783**	**81.51**	**41.59**	>20	2
	MMV1593534	38.51	79.54	5	0.5
	MMV1582493	42.42	8.14	10	1
	**MMV1581552**	**98.35**	**99.89**	>20	2
	MMV1580853	41.77	15.58	10	1
	MMV1579787	62.99	29.11	>20	2
	**MMV1581555**	**90.67**	**84.79**	>20	2
	MMV1580842	68.67	25.23	10	1
	MMV1580840	51.04	48.52	5	0.5
	MMV020752	82.04	0.72	1	0.1
	**MMV1229204**	**99.30**	**98.10**	>20	2
	MMV1578575	56.82	23.60	5	0.5
	MMV002354	17.27	72.25	10	1
	**MMV1578579**	**76.99**	**99.55**	10	1
	MMV1593532	7.11	74.89	10	1
	MMV002224	61.48	12.47	10	1
	MMV000051	79.69	24.68	>20	2
	**MMV687801**	**40.65**	**99.83**	≥20	2
	**MMV1579847**	**94.71**	**6.51**	≥20	2
	**MMV102270**	**96.99**	**99.48**	>20	2
	**MMV233495**	**92.11**	**80.34**	≥20	2
	MMV1633675	69.21	7.76	1	0.1
	MMV1582488	43.39	14.90	5	0.5
	MMV1580852	68.83	11.59	>20	2
	MMV1578891	49.02	1.73	>20	2
	**MMV1613563**	**84.04**	**NA**	20	2
Antifungal	MMV344625	41.90	4.84	>20	2
	**MMV1782108**	**85.01**	**86.38**	20	2
	MMV1634359	49.26	49.62	>20	2
Antiviral	MMV394033	74.65	2.71	20	2
	MMV1782208	43.78	NA	>20	2

a All the compounds selected for further stages are marked in bold. NA, No activity.

b Sub-MIC, 1/10 of the MIC value.

Compounds C1 to C4 significantly inhibited all the four tested QS-regulated extracellular virulence factors. Significant inhibition of LasA protease and LasB elastase was observed for compounds C3 and C4, which accounted for 15.01% and 21.62% of reduction, respectively (*P* < 0.0001) (Fig. 2B). Similarly, all the compounds inhibited the production of pyocyanin and its precursor phenazine-1-carboxylic acid (PCA) by P. aeruginosa by almost 50 to 70% compared to the controlled experiment (Fig. 2B, Fig. S2, and supplemental methods). The reduction in *lasR* and *rhlR* expression by these compounds can be correlated with the inhibition of extracellular virulence factors. Despite being one of the most extensively investigated antivirulence drug targets, *las* QS system-based inhibitors lack the lead-like properties required and so far have failed to proceed to preclinical development (20). However, our current work provides an opportunity for repositioning already approved drugs with preselected preference of high potency, low clogP, and avoidance of complex chemical structures (18). Since all the selected compounds reduce the virulence phenotypes, we also evaluated their ability to reduce biofilm formation. C1 to C4 were able to reduce the quantum dot (QD) fluorescence, similar to that of the positive control (Fig. 2C and D) corresponding to inhibition of biofilm formation. The high fluorescence observed in the negative control was due to the presence of a thick polysaccharide layer trapping QD (21). Biofilm created by bacterial growth affects QD mobility, and their thickness can be correlated with the fluorescence observed. Although we are unable to provide precise biofilm thickness, use of both positive and negative controls and their polarizing results assured us that the method can later be used for indirect measurement of biofilm thickness and/or viscosity. MMV1782108 (C1), a sulfonamide-containing antifungal drug showing antivirulence activity, may present an unexplored mode of action. Although this class of compounds has previously shown a wide range of antimicrobial activities (22), they have never been explored for their ability to inhibit receptors involved in the QS system and thus affecting the production of virulence factors. Three antibacterial compounds, MMV1579783 (C2), MMV1581552 (C3), and MMV1581555 (C4), were discovered to possess unique modes of action in inhibiting production of selected virulence factors, showing their interference in the hierarchical P. aeruginosa QS system and hinting toward their potential use in antivirulence therapy.

The ability to promptly respond to the rise in AMR has become of paramount importance given recent circumstances and triggered an intensive search for an alternative strategy. Our data provide an overview of one of the first approaches taken to use bioactive compound data sets such as the PRB and for repurposing known compounds and development of a drug with antivirulent potential. Together with other such efforts (18, 23, 24), our study shows the versatility of PRB-like compound data sets and the power of drug-repurposing strategies to combat AMR development as well as possibly other sudden medical emergencies. Well-described drug metabolism and pharmacokinetics (DMPK) profiles will provide an additional advantage for these compounds to rapidly progress through the drug discovery pipeline (18). Current work further highlights empirical evidence of targeting LasR and RhlR receptors from the QS cascade in P. aeruginosa toward the discovery of a new drug.

### Data availability.

We declare that all relevant data supporting the findings of this study are available within the paper and provided as supplemental methods, supplemental figures (Fig. S1 to S3), and a supplemental tables (Table S1).
