# Supplementary material for: Screening of the Medicines for Malaria Venture Pandemic Response Box for Discovery of Antivirulent Drug against Pseudomonas aeruginosa
Source: Microbiol Spectr. 2022 Oct 27;10(6):e02232-22. doi: 10.1128/spectrum.02232-22 (PMC9769688; doi:10.1128/spectrum.02232-22)
Supplement: Supplemental file 1 — Supplemental material. Download spectrum.02232-22-s0001.pdf, PDF file, 0.3 MB [file spectrum.02232-22-s0001.pdf]

Supplementary Information

Screening of MMV pandemic response box for discovery of antivirulent drug against *Pseudomonas aeruginosa*

Markéta Macho[a,b], Subhasish Saha[a], Grzegorz Konert[c], Avik Banerjee[c], Daniela Ewe[a] , Pavel Hrouzek[a], Petra Urajova[a] and Kumar Saurav[a#]

[a]Laboratory of Algal Biotechnology, Institute of Microbiology of the Czech Academy of Sciences - Center Algatech, Třeboň, Czech Republic.

[b]University of South Bohemia, Faculty of Science, České Budějovice, Czech Republic.

[c]Laboratory of Photosynthesis, Institute of Microbiology of the Czech Academy of Sciences - Center Algatech, Třeboň, Czech Republic.

Running Head: Antivirulent drug against *Pseudomonas aeruginosa*

#Address correspondence to Kumar Saurav, sauravverma17@gmail.com.

Table of Contents

1. Supplementary methods
2. Supplementary figure S1-S2
3. Supplementary Tables
4. Supplementary references

1. Supplementary methods

PRB stock solution: The Pandemic response box (PRB) containing 400 different compounds were kindly provided in 96-well microtiter plates containing 10 µl/well of 10 mM compound solutions in DMSO (1). All the compounds were diluted to obtain a final drug concentration of 1 mM in 25% DMSO in water. All the plates were stored at −20˚C until their use in the following experiments.

Quorum sensing inhibitory activity: The bioluminescence-based screening based on QSI assay was performed using two bioreporter strain; *E.coli* pSB401 (pSB401) and *E.coli* pSB1075 (pSB1075) reporters (2). Briefly, Biosensors pSB401 and pSB1075 were cultured overnight at 30°C with shaking (100 rpm) in LB medium (Luria Broth, HiMedia Laboratories, Mumbai, India) supplied with tetracycline and ampicillin to a final concentration of 15 µg/mL and 100 µg/mL respectively. The overnight cultures were exogenously supplemented with their cognate AHLs (Oxo-C_6-HSL and Oxo-C_{10}-HSL for pSB401 and pSB1075 respectively) to give a final concentration of 200 nM. 100 µL of the cultures were added into each well containing the tested compounds to reach the final concentration of 20 µM. The plates were sealed with parafilm and incubated at 30°C. Luminescence reading was performed after 4- and 6-hours using BMG POLAR star Omega Microplate Reader.

Activity of each fraction was calculated as mean percentage of decrease in bioluminescence emitted by the biosensor expressed as Relative Luminescence Units (RLU) relative to the average RLU value of negative control for each plate according to the formula given below:

$$Decrease\ (\%) = 100 - \left(\frac{100 \times mean\ of\ sample\ RLU}{mean\ of\ negative\ control\ RLU}\right)$$

Furanone F-30 (F30) were used as positive controls. The significance of differences between the mean values of tested subjects from their corresponding controls were tested using ANOVA Dunnett's test ($p < 0.05$) using GraphPad Prism software version 5.01. All the assays were performed in triplicates.

Determination of Minimum inhibitory concentration (MIC): Antimicrobial activity against *Pseudomonas aeruginosa* (CCM1959) was performed following the standard protocol of broth two-fold microdilution method (CLSI M100-S20) (3). Briefly, for broth microdilution method, the compounds were serially diluted at varying concentration of 20–0.03 µM in Mueller Hinton broth in 96-well plates. The inoculum (approximately 5×10^5 CFU/mL as final concentration) was prepared from an overnight culture of *P*. aeruginosa PAO1 and 0.1 mL of culture broth was added to each well. Plates were incubated aerobically at 37 °C for 16 h. Negative controls were prepared using culture media with DMSO (the dissolving solvent). Gentamycin (32–0.0625 µg/mL) was used as the positive control. After

incubation, the well with the lowest concentration of the compound showing some inhibition in the growth was taken as the MIC value. All the experiments were done in triplicates. Sub-MIC was determined as $1/10^{th}$ of the MIC.

Time-Kill Kinetics Test: In order to completely rule out any possibility of possessing inhibitory activity, a time-kill kinetics study was performed for all the four selected compounds (C1-C4). An inoculum containing a starting culture of bacteria with $1X10^6$ CFU/mL was added to the media and incubated at 37°C. Five different concentrations were used: 2X MIC, equal to MIC, MIC/2, MIC/4, and MIC/8. The colony forming unit (CFU) of the organisms was determined. The procedure was performed in triplicate (three independent experiments) and a graph of the log CFU/mL was plotted against time.

RNA isolation: *P. aeruginosa* PAO1 was grown in LB medium either with or without the tested compounds at sub-MIC concentrations (Table 1) for 40h at 37 °C. The cell pellets were collected by centrifugation at 13,000×g for 3 min at room temperature and were stored using DNA/RNA shield (Zymoresearch) at -80°C until further use.Total RNA was isolated using the Quick-RNA Fungal/Bacterial Miniprep Kit according to the manufacturer’s protocol (Zymo Research, Irvine, CA, USA). DNA-digestion treatment was performed to ensure that the RNA was free of any genomic DNA contamination. The RNA isolated was quantified using the Nanodrop spectrophotometer. The RNA samples were aliquoted and stored at −80 °C.

Gene expression analysis: Single-stranded cDNA was synthesised from total RNA using RevertAid first strand cDNA synthesis kit (ThermoFisher, Waltham, MA, USA). Using the cDNA as a template, realtime PCR amplification was done using CFX96 qPCR cycler (Bio-Rad Laboratories, Inc., Hercules, CA, USA). The final volume for each qPCR reaction was set for 25 µL, containing 400 nM of each primer and 1.5 µL of cDNA (synthetized in the previous step) in 12.5 µL of Maxima SYBR Green/ROX qPCR Master Mix (2X) (Thermo Scientific, USA). Following an initial 10 min denaturation/activation step at 95 °C, the mixture was subjected to 45 cycles of amplification including denaturation for 20 s at 95 °C; annealing for 20 s at 55 °C and extension for 30 s at 72 °C. Negative control in form of no template control were included to rule out any contamination during the preparation process. The melting curve of the amplified products was determined by setting up the program at 65 °C for 45 s followed by raising the temperature to 99 °C with 1 °C increment every 0.02 s. Threshold cycle value (Ct) was established for each reaction from the cycle number at which fluorescence was detectable over the threshold value calculated by Bio-Rad CFX Maestro software. The cutoff values for residual genomic DNA amplification and negative controls were set at greater than 35 and 40 cycles, respectively.

Relative gene expression data analysis: Data analysis was performed by simple ΔC_t method. Briefly, the arithmetic means of the C_t values of the technical replicates were calculated and then the data was normalised with the geometric mean of reference gene (rpoD and proC) to obtain ΔC_t values (4). The obtained output was then plotted together with the negative control experiment to evaluate the relative difference in the relative gene expression.

Antivirulence activity: The inhibition of extracellular virulence factors was tested for compounds C1-C4, and F-30 at their respective sub-MIC concentrations (Table 1) using *P. aeruginosa* PAO1 as described earlier (4). DMSO, solvent in which test compounds were dissolved, was used as negative control for both the experiments.

Inhibition of protease production: Protease produced by *Pseudomonas aeruginosa* was determined using the azocasein assay as previously described (5). Briefly, we treated *P. aeruginosa* PAO1 strain cultures with test compounds followed by incubation at 37 °C for 40 h. Cell free supernatant (30 µL) was transferred to a clean Eppendorf tube containing 100 µL of 1% azocasein (Sigma Aldrich, Czech Republic). The mixture was then incubated at 37 °C for 4 h. Reaction was arrested by the addition of 100 µL of 1.5 M HCl and subsequently, the mixture was incubated on ice for 30 min to precipitate the undigested azocasein. After the incubation, the mixture was centrifuged for 10 min at 15,000× *g* and supernatant were collected for further spectroscopic analysis. Prior to spectroscopic measurement at OD_{440}, 100 µL of 1 M NaOH was added. *P. aeruginosa* PAO1 without any treatment in diluting solvent (DMSO and sterile culture media) was used as the negative control.

Inhibition of elastase production: Culture supernatant (100 µL) was mixed with 200 µL of reaction buffer (100 mM Tris-HCl, 1 mM $CaCl_2$) containing the substrate elastin-Congo red (10 mg/mL) (Sigma Aldrich, Czech Republic). The reaction mixture was incubated for 3 h at 37 °C with shaking at 180 rpm. The reaction was terminated by adding 300 µL of 0.7 M sodium phosphate buffer (pH 6) and placing it on ice for 30 min. The absorbance of the supernatant was measured at 495 nm.

Relative quantification of Pyocyanin and its precursor phenazine-1-carboxylic acid: The production of pyocyanin (PYO) and its precursor phenazine-1-carboxylic acid (PCA) by *P. aeruginosa* PAO1 were quantified using HPLC analysis (6). The cell free supernatants were freeze dried, extracted with ethyl acetate (1 mL) and dried in vacuum. The dried crude extract was reconstituted in 500 µL of methanol for rapid detection of PYO and PCA.

Thermo Scientific DionexUltiMate 3000 UHPLC (Thermo Scientific) equipped with a diode array detector (DAD) was used for analysis of the crude extract. HPLC separation was performed on reversed phase Kinetex Phenomenex C_{18} column (150 X 4.6 mm, 2.6 µm) with H_2O/ CH_3CN containing 0.1% HCOOH as a mobile phase. Flow rate during

analysis was 0.6 mL/min. The gradient was as follows: H_2O/ CH_3CN 85/15 (0 min), 85/15 (in 1 min), 0/100 (in 20 min), 0/100 (in 25 min) and 85/15 (in 30 min). HPLC was connected to a high-resolution mass spectrometry with electrospray ionization source (ESI-HRMS; Impact HD Mass Spectrometer, Bruker). Mass spectrometer settings were as follows: dry temperature 200 °C; drying gas flow 12 L/min; nebulizer 3 bar; capillary voltage 4500 V; endplate offset 500 V. The spectra were collected in the range 20-700 *m/z* with spectra rate 2 Hz. MS/MS acquisition of PYO and PCA was operated in the same parameter using collision energy of 12.8 eV and 13.1 eV respectively in positive mode.

Static biofilm assay: Biofilm thickness was measured indirectly by applying quantum dots (QD) solutions onto the biofilms. *P. aeruginosa* PAO1 biofilms were grown on 15 µ-Slide $VI^{0.4}$ IBIDI slides (ibidi Gmbh, Germany) statically for 40 h at 37 °C with or without the test compounds (C1-C4), and F-30 at sub-MIC concentrations. DMSO, solvent in which test compounds were dissolved was used as negative control. Reservoirs in the slide were repeatedly filled to avoid any drying of slide channel (30 µL) forming biofilm. After the incubation, planktonic culture was pipetted out and was repeatedly washed with the LB medium to remove any unattached free moving cells.

Microscope image acquisition and analysis

Channel was further washed with QD solution (Fluoro-Max Blue B150, 1:5 dilution in LB medium (90 µL final volume). After 10 min of incubation, that allowed QD to penetrate through whole length of channel evenly, plate was placed on Confocal microscope stage (Zeiss LSM 880; Carl Zeiss Microscopy GmbH) (7). Images were taken at 2 spots for each sample's channel: in the middle point between two wells and in between middle point and the upper application well. This was used to check if QD penetration is distance-related to the application point. Each image was taken as the Z-stack composed of 90 slices each positioned in 200 nm intervals, with middle slice being set to the beginning of the glass plate. Objective: C-Apochromat 40x/1.2 W Korr FCS M27. Laser: 405 nm, laser power: 0.03%, detection: 464-490 nm, gain: 800, dwell time: 1.3 us, MBS: -405, size 512x512 pix (42.5 x 24.5 um), 8-bit mode. Images were done in such a way that several last slices were placed way above polysaccharide layer, thus containing only free floating QD and bacteria. Each image was separated into 4x4 grid to create 16 sub-images. Each of the sub-images was analysed independently. Fluorescence emissions were extracted using ImageJ (1.53p) with Bio-Formats plugin (6.9.0). The statistical analysis was carried out with R 3.6.2 in Rstudio 1.2.5033. Packages used: ggplot (8). Data were analysed with one-way ANOVA followed up with post hoc Tukey test. All significance points ($p < 0.05$) of interest were calculated against NC sample.

2. Supplementary figures

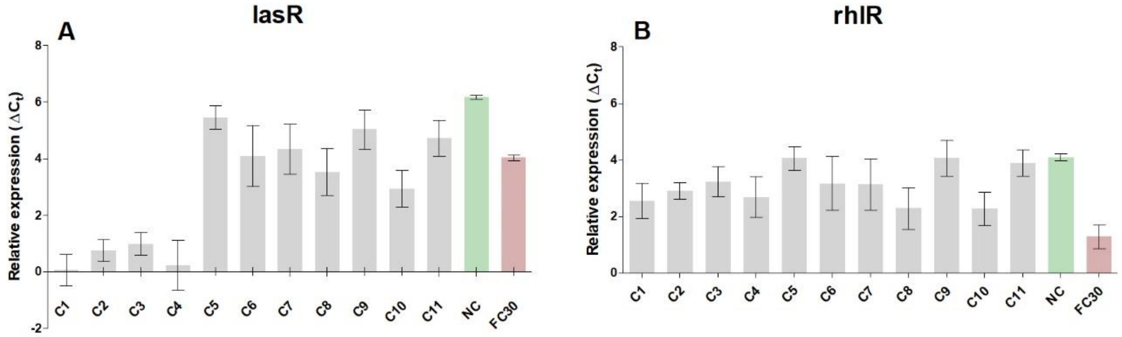

Fig. S1. Transcriptional expression of QS regulatory genes lasR (A) and rhlR (B). Expression levels were quantified by RT-qPCR, relative expression in terms of ΔC_t are plotted for target genes and were normalised to the geometric mean of two reference genes (rpoD and proC). Vertical bars represent S.D. ± (n = 3).

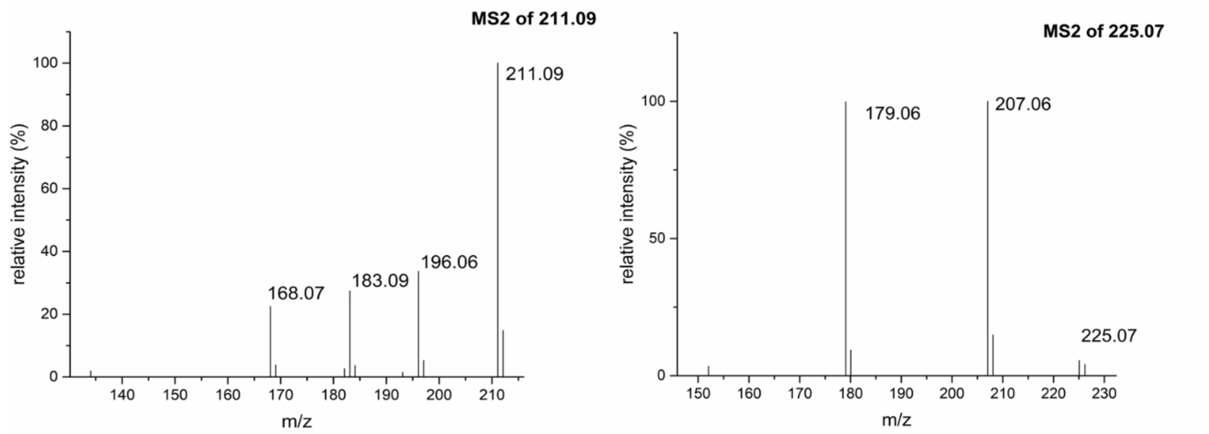

Fig. S2. HR-MS/MS product ion spectra of protonated molecule $[M + H]^+$) at *m/z* 211.09 (Pyocyanin) and *m/z* 225.07 (phenazine-1-carboxylic acid) derived from the cell free extract of *P. aeruginosa* PAO1.

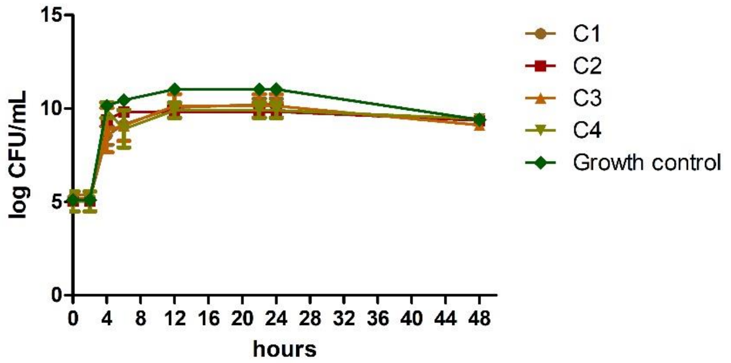

Fig. S3 Time-kill curves with and without addition of compounds (C1-C4) at 2X MIC (40µM).

3. Supplementary Tables

Table S1: Toxicity and antimicrobial activity of selected compounds (C1-C4).

Compounds	Cytotoxicity (μM)	Activity against ESKAPE pathogens (μM)						Activity against other pathogens (μM)
		Enterococcus faecium	***Staphylococcus aureus***	***Klebsiella pneumoniae***	***Acinetobacter baumanii***	***Pseudomonas aeruginosa***	***Enterobacter spp.***	
C1	HepG2, **LD$_{50}$ at >1000**	ND	ND	ND	ND	ND	ND	ND
C2	Human RBC, **HC$_{10}$ at 20** HEK293 cells, **CC$_{50}$ at 20**	ND	*Staphylococcus aureus* MRSA **MIC at 20**	ATCC 70063 **MIC at 20**	ATCC 19606 **MIC at 2**0	PA01 **MIC at 20** ATCC 27853 **MIC at 20**	ND	*Enterococcus faecalis* murI **IC$_{50}$ 2.4**
C3	HEK293 cells, **CC$_{50}$ at 20** Human RBC, **HC$_{10}$ at 20**	ND	MRSA ATCC 43300 **MIC at 20**	ND	ATCC 19606 **MIC at 20**	PAO397 **MIC at 20**	ND	*Escherichia coli,* **IC$_{50}$ at 6.6**
C4	HEK293 cells, **CC$_{50}$ at 20** Human RBC, **HC$_{10}$ at 20**	ND	MRSA ATCC 43300 **MIC 20**	ATCC 70063, **MIC at 20**	ATCC 19606 **MIC at 20**	PAO397, **MIC at 20** ATCC 27853 at **MIC 20**	ND	*E. coli* ATCC 25922, **MIC at 20**

Note: ND stands for not determined

4. Supplementary references:

1. Samby K, Besson D, Dutta A, Patra B, Doy A, Glossop P, Mills J, Whitlock G, Hooft van Huijsduijnen R, Monaco A, Bilbe G, Mowbray C, Perry B, Adam A, Wells TNC, Willis PA. 2022. The Pandemic Response Box─Accelerating Drug Discovery Efforts after Disease Outbreaks. ACS Infectious Diseases 8:713-720.
2. Saurav K, Costantino V, Venturi V, Steindler L. 2017. Quorum Sensing Inhibitors from the Sea Discovered Using Bacterial N-acyl-homoserine Lactone-Based Biosensors. Marine Drugs 15.
3. Saurav K, Macho M, Kust A, Delawska K, Hajek J, Hrouzek P. 2019. Antimicrobial activity and bioactive profiling of heterocytous cyanobacterial strains using MS/MS-based molecular networking. Folia Microbiol (Praha) 64:645-654.
4. Ahmed S, Rudden M, Smyth TJ, Dooley JSG, Marchant R, Banat IM. 2019. Natural quorum sensing inhibitors effectively downregulate gene expression of *Pseudomonas aeruginosa* virulence factors. Appl Microbiol Biotechnol 103:3521-3535.
5. Costantino V, Della Sala G, Saurav K, Teta R, Bar-Shalom R, Mangoni A, Steindler L. 2017. Plakofuranolactone as a Quorum Quenching Agent from the Indonesian Sponge *Plakortis* cf. lita. Marine Drugs 15.
6. Kushwaha M, Jain SK, Sharma N, Abrol V, Jaglan S, Vishwakarma RA. 2018. Establishment of LCMS Based Platform for Discovery of Quorum Sensing Inhibitors: Signal Detection in *Pseudomonas aeruginosa* PAO1. ACS Chem Biol 13:657-665.
7. Elzorkany HE, Farghali MA, Hassan MA, El-Sayed K, Canonico M, Konert G, Farroh K, Elshoky HA, Kana R. 2019. Ecotoxicology impact of silica-coated CdSe/ZnS quantum dots internalized in *Chlamydomonas reinhardtii* algal cells. Sci Total Environ 666:480-489.
8. Wickham H. 2016. ggplot2, 2 ed doi:https://doi.org/10.1007/978-3-319-24277-4. Springer Cham.
